# Population genomics of American mink using genotype data

**DOI:** 10.3389/fgene.2023.1175408

**Published:** 2023-05-09

**Authors:** Guoyu Hu, Duy Ngoc Do, Ghader Manafiazar, Alyson A. Kelvin, Mehdi Sargolzaei, Graham Plastow, Zhiquan Wang, Younes Miar

**Affiliations:** ^1^ Department of Animal Science and Aquaculture, Dalhousie University, Truro, NS, Canada; ^2^ Vaccine and Infectious Disease Organization (VIDO), University of Saskatchewan, Saskatoon, SK, Canada; ^3^ Department of Pathobiology, University of Guelph, Guelph, ON, Canada; ^4^ Select Sires Inc, Plain City, OH, United States; ^5^ Livestock Gentec, Department of Agricultural, Food and Nutritional Science, University of Alberta, Edmonton, AB, Canada

**Keywords:** American mink, effective population size, linkage disequiblibrium, population genomics, genotypes

## Abstract

Understanding the genetic structure of the target population is critically important to develop an efficient genomic selection program in domestic animals. In this study, 2,973 American mink of six color types from two farms (Canadian Centre for Fur Animal Research (CCFAR), Truro, NS and Millbank Fur Farm (MFF), Rockwood, ON) were genotyped with the Affymetrix Mink 70K panel to compute their linkage disequilibrium (LD) patterns, effective population size (*Ne*), genetic diversity, genetic distances, and population differentiation and structure. The LD pattern represented by average *r* ^2^, decreased to <0.2 when the inter-marker interval reached larger than 350 kb and 650 kb for CCFAR and MFF, respectively, and suggested at least 7,700 and 4,200 single nucleotide polymorphisms (SNPs) be used to obtain adequate accuracy for genomic selection programs in CCFAR and MFF respectively. The *Ne* for five generations ago was estimated to be 76 and 91 respectively. Our results from genetic distance and diversity analyses showed that American mink of the various color types had a close genetic relationship and low genetic diversity, with most of the genetic variation occurring within rather than between color types. Three ancestral genetic groups was considered the most appropriate number to delineate the genetic structure of these populations. Black (in both CCFAR and MFF) and pastel color types had their own ancestral clusters, while demi, mahogany, and stardust color types were admixed with the three ancestral genetic groups. This study provided essential information to utilize the first medium-density SNP panel for American mink in their genomic studies.

## 1 Introduction

American mink (*Neogale vison*) is a semiaquatic and carnivorous mammal that belongs to the weasel (*Mustelidae*) family (García et al., 2010). It is native to North America but has been farmed in many countries and used as one of the primary fur sources for fur industries worldwide due to its high-quality fur and various colors (Anistoroaei et al., 2009; Tamlin et al., 2009; Thirstrup et al., 2015; Zhang L. et al., 2021). With the COVID-19 (coronavirus disease from 2019) pandemic and the market downturn, the mink industry faces serious challenges. In Canada, from 2015 to 2020, the number of mink farms dropped from 213 to 63, decreasing mink production from three million to one million per year (Statistics Canada, 2022). However, the mink industry appears to be on the upturn, as market demand and fur prices have increased, based on fur auction reports in recent years (Oaten, 2021; sagafurs, 2022). With a smaller number of mink farms, improving the efficiency (e.g., improved disease resilience, feed efficiency, reproduction performance, and pelt quality) of mink farms through advanced genomic selection programs could help to meet the rising market demand and help mink farmers obtain more economic benefits from the rising pelt prices. Genomic selection has been applied in the main livestock species, such as dairy cattle (Wiggans et al., 2017), swine (Miar et al., 2015; Knol et al., 2016), and poultry (Wolc et al., 2016), to improve genetic merit, but this breeding strategy has not been utilized in the mink industry to date.

To develop an efficient genomic selection program in domestic animals, understanding the genetic structure of the target population is essential (Groeneveld et al., 2010; Wellmann and Bennewitz, 2019). American mink of different color types show different performance for some traits. For example, it has been known that light-colored mink are more susceptible to the Aleutian mink disease virus than dark color types (Ellis, 1996). Meanwhile, better reproductive performance was observed in brown color mink compared with the other color types (Kidd et al., 2009). Thus, investigating the genetic structure of American mink of various color types could also help explain variation in performance for traits of economic interest. The genetic structure of target populations is usually revealed by exploring domestication history, genetic diversity, genetic relationship, and genetic pattern of the populations. Linkage disequilibrium (LD) and effective population sizes (*Ne*) are two important parameters for revealing genetic structure of target population. The LD is defined as the non-random association of alleles at two or more loci (Slatkin, 2008). Genetic drift, selection, epistatic combinations, population structure, and admixture between distinct populations are all potential causes leading to LD between unlinked markers (Pfaff et al., 2001; Ardlie et al., 2002; Qanbari, 2020). The magnitude of LD is used to determine the appropriate density of markers for genome-wide mapping studies (Goddard and Hayes, 2009), and both genomic selection and genome-wide association studies (GWAS) depend on the presence of LD between markers and functional variants (Bush and Moore, 2012; Hay and Rekaya, 2018). In the meantime, the extent of LD between unlinked loci can be utilized to estimate the recent and past *Ne* (Hill, 1981; Waples and Do, 2010). The *Ne* is used to measure the rate of inbreeding and loss of genetic diversity and quantify the extent of variability in a population and the effectiveness of selection relative to drift (Charlesworth, 2009; Ryman et al., 2019). Analysis of molecular variance (AMOVA) is another popular method of detecting population differentiation (Excoffier et al., 1992). The AMOVA can explain the genetic variation patterns of studied populations by quantifying the contribution of various population structure levels using marker data from different genotypes (Fitzpatrick, 2009). In addition to AMOVA, discriminant analysis of principal components (DAPC) (Jombart et al., 2010) and ADMIXTURE (Alexander et al., 2009) are also common analyses used to assess the genetic structure of a population using molecular marker information. In brief, DAPC is a multivariate method that can identify and describe clusters of individuals which are genetically related (Jombart et al., 2010; Deperi et al., 2018; Thia, 2022), and ADMIXTURE can infer the number of ancestral populations that generated the current population and the proportions of individual genomes derived from each ancestral population (Alexander et al., 2009; Alexander and Lange, 2011; Liu et al., 2020).

The genetic structures of farm and feral American mink were previously studied using information from different molecular markers, including microsatellite, mitochondrial DNA, and single nucleotide polymorphism (SNP) markers. Microsatellite loci were used to investigate the genetic structures of wild-caught American mink in Japan (Yukari et al., 2010), Sweden (Zalewski et al., 2016), and Spain (Lecis et al., 2008). The information from mitochondrial DNA and 11 microsatellite loci were applied to understand the genetic structure of introduced American mink in southern Chile (Mora et al., 2018). Genotypes obtained from 194 SNPs, generated from the restriction-site associated DNA sequencing method, were used to investigate the population genetic structure of farm and feral American mink in Poland and Denmark (Thirstrup et al., 2015). Data containing 13,321 SNPs, which were detected using the genotyping-by-sequencing (GBS) approach on 46 scaffolds from 285 black American mink, were used to investigate LD and *Ne* of black American mink in Canada (Karimi et al., 2020). Moreover, 100,000 SNPs, which were randomly selected through whole genome sequencing (WGS) across 51 scaffolds from 100 farm mink, were used to investigate the genetic structure of American mink in Canada (Karimi et al., 2021). However, there is no study investigating the genetic structure of farmed American mink with various color types using a relatively large sample size (about 3,000) with genotypic data from a medium-density SNP panel.

Investigation of the genetic structure of American mink using genotypic data from a medium-density SNP panel will benefit the future use of this genotyping panel for use in genomic selection, as well as other genomic studies, such as quantitative trait locus mapping, identification of signatures of selection, and GWAS. Meanwhile, one critical factor affecting the accuracy of estimating population genetic diversity parameters is the sample size (Bashalkhanov et al., 2009). The sample size in the previous studies, which investigated the genetic structure of American mink, were all less than 300 individuals (Lecis et al., 2008; Yukari et al., 2010; Thirstrup et al., 2015; Zalewski et al., 2016; Mora et al., 2018; Karimi et al., 2020; Karimi et al., 2021). Small sample sizes could lead to significant errors in determining allelic richness and therefore influence the accuracy of the estimators of genetic diversity in populations (Bashalkhanov et al., 2009). Thus, the main purpose of this study was to use genotypic data from the first medium-density 70K SNP panel for American mink with a larger sample size to 1) investigate the LD pattern and *Ne* of farm American mink in Canada, 2) explore the genetic distance and genetic diversity among various color types of American mink, and 3) reveal the genetic structure and admixture pattern of farm American mink in Canada.

## 2 Materials and methods

### 2.1 Ethics statement

This study was approved by the Dalhousie University Animal Care and Use Committee (certification#: 2018-009 and 2019-012). All the mink were raised based on the Code of Practice for the Care and Handling of Farmed Mink guidelines from the Canada Mink Breeders Association (NFACC, 2013).

### 2.2 Animals and sampling

The individuals used in this study were from two farms, including the Canadian Center for Fur Animal Research (CCFAR, *n* = 1,411) at Dalhousie University, Faculty of Agriculture (Nova Scotia, Canada) and Millbank Fur Farm (MFF, *n* = 1,562) at Rockwood (Ontario, Canada). Mink from CCFAR included five color types: black (CBL, *n* = 177), demi (CDE, *n* = 542), mahogany (CMA, *n* = 527), pastel (CPA, *n* = 152), and stardust (CST, *n* = 13). The colors of the studied mink were identified and assigned to them at their weaning age by experienced technicians at CCFAR. All individuals from MFF were Black color type (MBL, *n* = 1,562). There was no migration of mink between the two farms. There was no regular mating system on both farms, and breeders were selected based on their phenotypic performances without considering the color types.

### 2.3 Sample collection and genotyping

DNA extraction was performed on tongue tissue from animals using the DNeasy Blood and Tissue Kit (Qiagen, Hilden, Germany), according to the manufacturer’s instructions. The quantity and quality of DNA were measured with a NanoDrop ND-1000 spectrophotometer (NanoDrop Technologies Inc., Wilmington, DE). The 260/280 nm readings for all samples ranged from 1.8 to 2.0. All samples were diluted to a final concentration of 500 ng, checked for DNA quality, and finally genotyped by Axiom Affymetrix Mink 70K panel (Neogen, Lincoln, Nebraska, United States) (Do et al., 2023).

### 2.4 Animals and SNP quality control

Prior to analyses of the genotyping data, animals and SNPs were excluded from the dataset based on the following criteria using PLINK software (Purcell et al., 2007): SNPs having a minor allele frequency lower than 1%, a call rate lower than 90%, an excess of heterozygosity higher than 15%, and Mendelian error frequency larger than 5%, SNPs that were out of Hardy-Weinberg equilibrium with very low probability (1 × 10^−5^), and individuals with a call rate lower than 90%. Overall, 2,973 genotyped animals with 24,161 SNPs remained for the following analyses.

### 2.5 Population genetic parameters, linkage disequilibrium, and effective population size

The average minor allele frequency (MAF) and observed heterozygosity were estimated for each color type and whole CCFAR population using SNP1101 software (Sargolzaei, 2014). The nucleotide diversity was conducted for each SNP and color type and whole CCFAR population based on the method proposed by Nei and Li (1979) using VCFtools software (Danecek et al., 2011).

Linkage disequilibrium (*r* ^2^) was measured as proposed by Hill and Robertson (1968) and calculated according to the following equation using SNP1101 software (Sargolzaei, 2014):

$$r_{ij}^{2}=\frac{{\left(P_{ij}-P_{i}P_{j}\right)}^{2}}{P_{i}∙\left(1-P_{i}\right)∙P_{j}\left(1-P_{j}\right)}$$

in which P_*ij*_ is the frequency of the two-marker haplotype (*i* = allele *i* at locus 1; *j* = allele *j* at locus 2), and P_*i*_ and P_*j*_ are the frequencies of allele *i* at locus 1 and allele *j* at locus 2, respectively (Badke et al., 2012).

The LD was calculated in four distance sets with different bin sizes, which included 100 kb with a bin size of 10 kb, 500 kb with a bin size of 50 kb, 1,000 kb with a bin size of 100 kb, and 10 Mb with a bin size of 1,000 kb. The average *r* ^2^ of each bin was plotted against the median size of the bin to show the trend of LD with the increases in genome distances.

Effective population sizes for various color types were estimated using SNP1101 software (Sargolzaei, 2014) by the following equation (Sved, 1971):

$$Ne=\left(\frac{1}{4c}\right)\left(\frac{1}{r^{2}}-1\right)$$

in which *Ne* is the effective population size; c is the marker distance in Morgans. Additionally, past effective population size at generation T was calculated by the approximation T = $\frac{1}{2c}$ (Hayes et al., 2003). Effective population size was calculated for 1, 5, 10, 20, 50, 100, 200, and 250 generations ago.

### 2.6 Genetic distances and genetic diversity

Pairwise genetic distances were calculated using Nei’s (Nei, 1972) method (standard genetic distance method) under the “StAMPP” package of R (Pembleton et al., 2013). Additionally, a dendrogram of genetic distance among all color types was produced through the unweighted pair group method with the arithmetic mean method in the “poppr” R package (Kamvar et al., 2014) based on Nei’s distance (Nei, 1972). The pairwise Fst was calculated based on Weir and Cockerham’s procedures (Weir and Cockerham, 1984) using the “StAMPP” package of R (Pembleton et al., 2013). The Nei’s genetic distances matrix of the six color types was also used to construct the phylogenetic trees using the unweighted pair group method in the “poppr” R package (Kamvar et al., 2014). In addition, AMOVA (Excoffier et al., 1992) was performed using ade4 implemented in the “poppr” R package (Kamvar et al., 2014) to determine the partition of genetic diversity among samples at different hierarchical levels.

### 2.7 Genetic structure and admixture patterns

Population structure was analyzed by the discriminant analysis of principal components (DAPC) method using the “adegenet” package of R (Jombart et al., 2010). The number of clusters in the population was defined by using the *find. clusters* function under the “adegenet” package. This function implements a clustering procedure used in DAPC by running successive K-means with an increasing number of clusters (K) after transforming data using a principal component analysis (PCA). The most suitable number of clusters has the lowest associated Bayesian Information Criterion (BIC). An α-score optimization was used to determine the number of principal components to retain. Additionally, an unsupervised analysis in ADMIXTURE version 1.3.0 (Alexander et al., 2009) was applied to further assessing the potential admixture among the various color types. Five-fold cross-validation (CV) procedure was performed, and the CV scores were used to determine the best K value.

## 3 Results

### 3.1 Population genetic parameters, linkage disequilibrium and effective population size

The MAF, observed heterozygosity, and nucleotide diversity for each SNP, color type and whole CCFAR population are present in Table 1. The average MAF ranged from 0.212 (mahogany) to 0.246 (stardust). The lowest level of observed heterozygosity (30.6%) was observed in mahogany color type of CCFAR, whereas the stardust color type of CCFAR had the highest percentage of observed heterozygosity (34.9%). The overall nucleotide diversity ranged from 0.283 (stardust) to 0.307 (demi). Considering the whole CCFAR population individuals, the MAF was 0.216, the observed heterozygosity was 30.576%, and the overall nucleotide diversity was 0.307 (Table 1).

**TABLE 1:** Average minor allele frequency (MAF), observed heterozygosity, and nucleotide diversity for five color types of American mink in CCFAR, whole CCFAR population, and whole MFF population.

Color type	Number of individuals	Average MAF	Observed heterozygosity (%)	Average nucleotide diversity
CBL^a^	177	0.220	31.821	0.297
CDE^b^	542	0.216	31.029	0.307
CMA^c^	527	0.212	30.594	0.303
CPA^d^	152	0.236	32.644	0.294
CST^e^	13	0.246	34.947	0.283
CCFAR^f^	1,411	0.216	30.576	0.307
MBL^g^	1,562	0.226	31.938	0.288

^a^Black color type mink in the canadian center for fur animal research (CCFAR).

^b^ Demi color type mink in CCFAR.

^c^ Mahogany color type mink in CCFAR.

^d^ Pastel color type mink in CCFAR.

^e^ Stardust color type mink in CCFAR.

^f^ All mink in CCFAR.

^g^ Black color type mink in Millbank Fur Farm.

**FIGURE 1:**
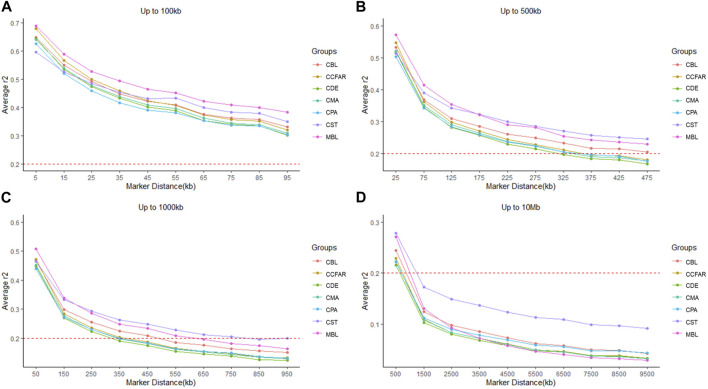
Linkage disequilibrium measured by r^2^ plotted as a function of inter-market distance (kb) **(A)** from 0 up to 100 kb using consecutive 10 kb bins, **(B)** up to 500 kb using consecutive 50 kb bins, **(C)** up to 1,000 kb using consecutive 100 kb bins, and **(D)** up to 10 Mb using consecutive 1,000 kb bins. CCFAR = all mink from the Canadian Center for Fur Animal Research (CCFAR). MBL = black color type mink in Millbank Fur Farm; CBL, CDE, CMA, CPA, and CST are black, demi, mahogany, pastel, and stardust color type mink in CCFAR, respectively.

The average *r* ^2^ between adjacent SNPs on all chromosomes for six color types of American mink and the whole CCFAR American mink population are presented in Table 2. The average *r* ^2^ between adjacent SNPs among various color types ranged from 0.373 ± 0.402 (CPA) to 0.406 ± 0.408 (MBL). Compared with the MFF population (MBL), the average *r* ^2^ between adjacent SNPs for the whole CCFAR population (0.399 ± 0.404) was lower (Table 2). The LD decay measured by *r* ^2^ with different inter-marker distances (up to 100 kb, 500 kb, 1,000 kb, and 10 Mb) and consecutive bins (10 kb, 50 kb, 100 kb, and 1 Mb) in six color types mink is presented in Figure 1. Within the 1,000 kb inter-marker distance range, MBL and CST showed the two highest average *r* ^2^ among all color types at the same inter-marker distance, while CDE and CPA had the two lowest average *r* ^2^ among all color types. CDE reached an average *r* ^2^ < 0.2 with the shortest inter-marker distance (around 325 kb) among all color types, while CST reached an average *r* ^2^ < 0.2 with the longest inter-marker distance (around 850 kb) among all color types. Both CMA and CPA reached an average *r* ^2^ < 0.2 at the inter-marker distance of approximately 350 kb. CBL and MBL reached an average *r* ^2^ < 0.2 at the inter-marker distance of approximately 475 kb and 650 kb, respectively. The average *r* ^2^ of the whole CCFAR population was <0.2 at the inter-marker distance of 350 kb. The most rapid LD decays for all color types were observed when the average inter-marker distances increased from 50 to 150 kb, and CDE had the most rapid reduction of LD in this interval (Figure 1).

**TABLE 2:** Summary of the average and standard deviation of *r* ^2^ between adjacent SNPs on all chromosomes five color types of American mink in CCFAR, whole CCFAR population, and whole MFF population.

	CCFAR^b^	CBL^c^	CDE^d^	CMA^e^	CPA^f^	CST^g^	MBL^h^
Chr^a^	Average *r* ^2^±SD	Average *r* ^2^±SD	Average *r* ^2^±SD	Average *r* ^2^±SD	Average *r* ^2^±SD	Average r2±SD	Average *r* ^2^±SD
1	0.444 ± 0.451	0.403 ± 0.445	0.425 ± 0.447	0.415 ± 0.445	0.415 ± 0.441	0.408 ± 0.438	0.419 ± 0.451
2	0.412 ± 0.446	0.379 ± 0.443	0.395 ± 0.440	0.399 ± 0.441	0.387 ± 0.435	0.369 ± 0.424	0.392 ± 0.445
3	0.354 ± 0.440	0.336 ± 0.431	0.339 ± 0.433	0.340 ± 0.434	0.335 ± 0.425	0.346 ± 0.420	0.341 ± 0.429
4	0.404 ± 0.447	0.396 ± 0.449	0.385 ± 0.443	0.384 ± 0.442	0.367 ± 0.434	0.400 ± 0.440	0.406 ± 0.450
5	0.369 ± 0.365	0.368 ± 0.369	0.346 ± 0.364	0.356 ± 0.365	0.336 ± 0.366	0.368 ± 0.378	0.388 ± 0.379
6	0.432 ± 0.453	0.391 ± 0.445	0.418 ± 0.448	0.415 ± 0.449	0.404 ± 0.442	0.372 ± 0.430	0.422 ± 0.447
7	0.396 ± 0.377	0.393 ± 0.381	0.380 ± 0.378	0.381 ± 0.377	0.375 ± 0.380	0.396 ± 0.383	0.407 ± 0.385
8	0.428 ± 0.378	0.425 ± 0.388	0.412 ± 0.381	0.423 ± 0.382	0.403 ± 0.386	0.382 ± 0.389	0.449 ± 0.392
9	0.345 ± 0.351	0.357 ± 0.360	0.334 ± 0.351	0.342 ± 0.355	0.331 ± 0.355	0.361 ± 0.371	0.357 ± 0.362
10	0.269 ± 0.397	0.268 ± 0.399	0.261 ± 0.390	0.248 ± 0.385	0.263 ± 0.384	0.285 ± 0.387	0.277 ± 0.404
11	0.558 ± 0.386	0.557 ± 0.399	0.531 ± 0.393	0.540 ± 0.391	0.502 ± 0.406	0.533 ± 0.432	0.597 ± 0.397
12	0.504 ± 0.377	0.519 ± 0.394	0.470 ± 0.378	0.499 ± 0.387	0.438 ± 0.387	0.490 ± 0.419	0.545 ± 0.397
13	0.384 ± 0.382	0.397 ± 0.386	0.364 ± 0.380	0.376 ± 0.381	0.368 ± 0.381	0.416 ± 0.396	0.427 ± 0.396
14	0.293 ± 0.401	0.291 ± 0.402	0.266 ± 0.390	0.285 ± 0.393	0.293 ± 0.399	0.340 ± 0.421	0.258 ± 0.372
Mean	0.399 ± 0.404	0.391 ± 0.407	0.380 ± 0.401	0.386 ± 0.402	0.373 ± 0.402	0.390 ± 0.409	0.406 ± 0.408

^a^ Chromosome.

^b^ All mink from the Canadian Center for Fur Animal Research (CCFAR).

^c^ Black color type mink in CCFAR.

^d^ Demi color type mink in CCFAR.

^e^ Mahogany color type mink in CCFAR.

^f^ Pastel color type mink in CCFAR.

^g^ Stardust color type mink in CCFAR.

^h^ Black color type mink in Millbank Fur Farm.

*r* ^2^ = Linkage disequilibrium; SD, standard deviation.

**FIGURE 2:**
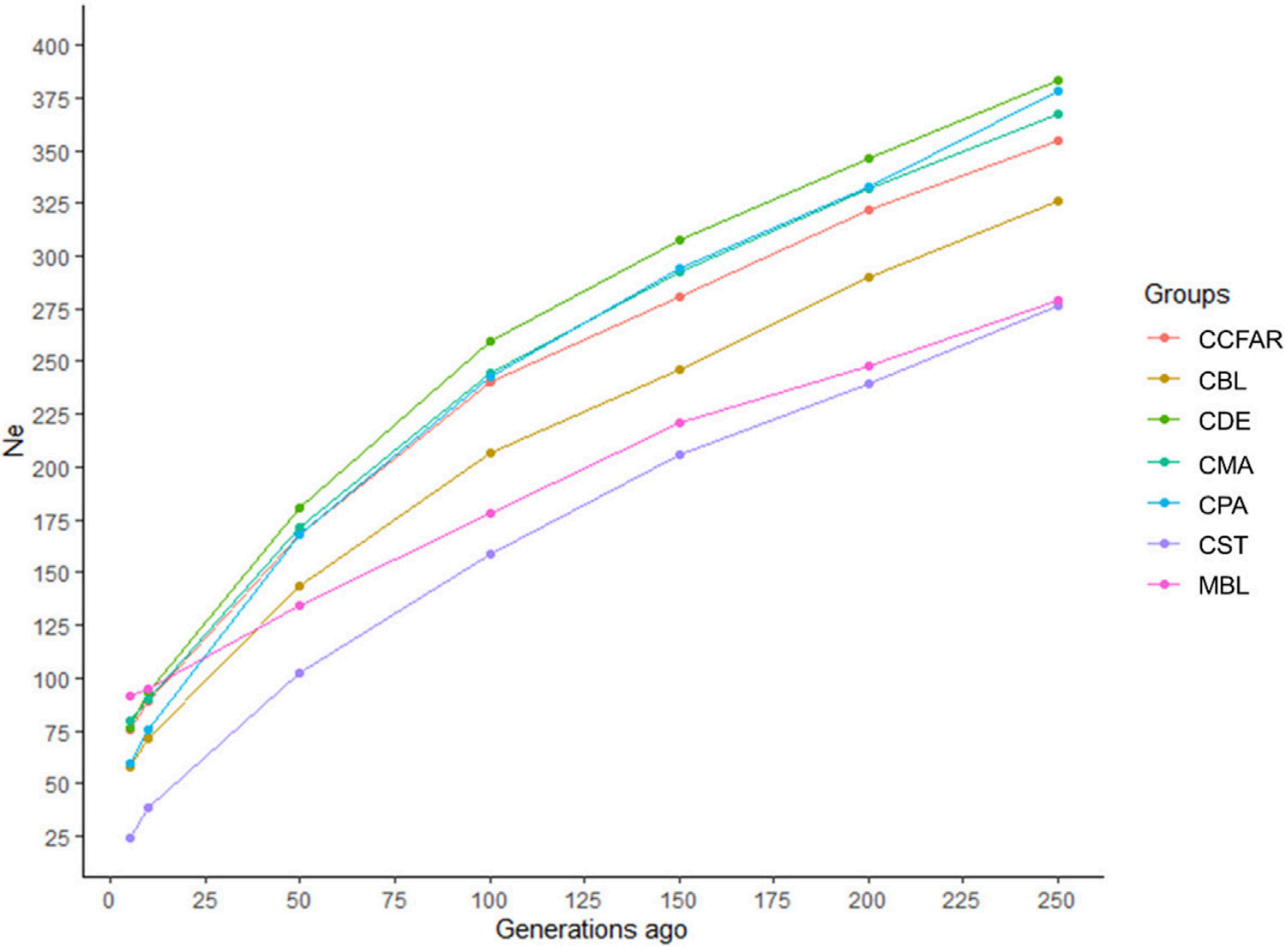
Estimated effective population sizes for various color types of American mink from 5 to 250 generations ago. CCFAR = all mink from the Canadian Center for Fur Animal Research (CCFAR). MBL = black color type mink in Millbank Fur Farm; CBL, CDE, CMA, CPA, and CST are black, demi, mahogany, pastel, and stardust color type mink in CCFAR, respectively.

The *Ne* was evaluated based on LD estimates (*r* ^2^) from five to 250 generations ago, and the estimates of *Ne* are shown in Figure 2. In general, all the color types showed a marked decrease over generations. The recent *Ne*, five generations ago, of CBL, CDE, CMA, CPA, CST, and MBL was 58, 76, 80, 60, 24, and 91, respectively. For the whole CCFAR population, the *Ne* was 76 at five generations ago. The maximum *Ne* was observed 250 generations ago for all color types, where CDE had the highest *Ne* of 384 and CST had the lowest *Ne* of 276. CDE had the highest *Ne*, and CST had the lowest *Ne* from 50 to 250 generations ago. However, in more recent generations, from five to ten generations ago, MBL was observed to have the highest *Ne*, and CST was found to have the lowest *Ne*. The decline of *Ne* was more rapid from 5 to 50 generations ago for all color types in CCFAR and from 5 to 100 generations ago for the MFF population. In the meantime, in more recent generations (5–50 generations ago), the *Ne* of the CCFAR population decreased more rapidly than the MFF population (Figure 2).

### 3.2 Genetic distances and genetic diversity

**FIGURE 3:**
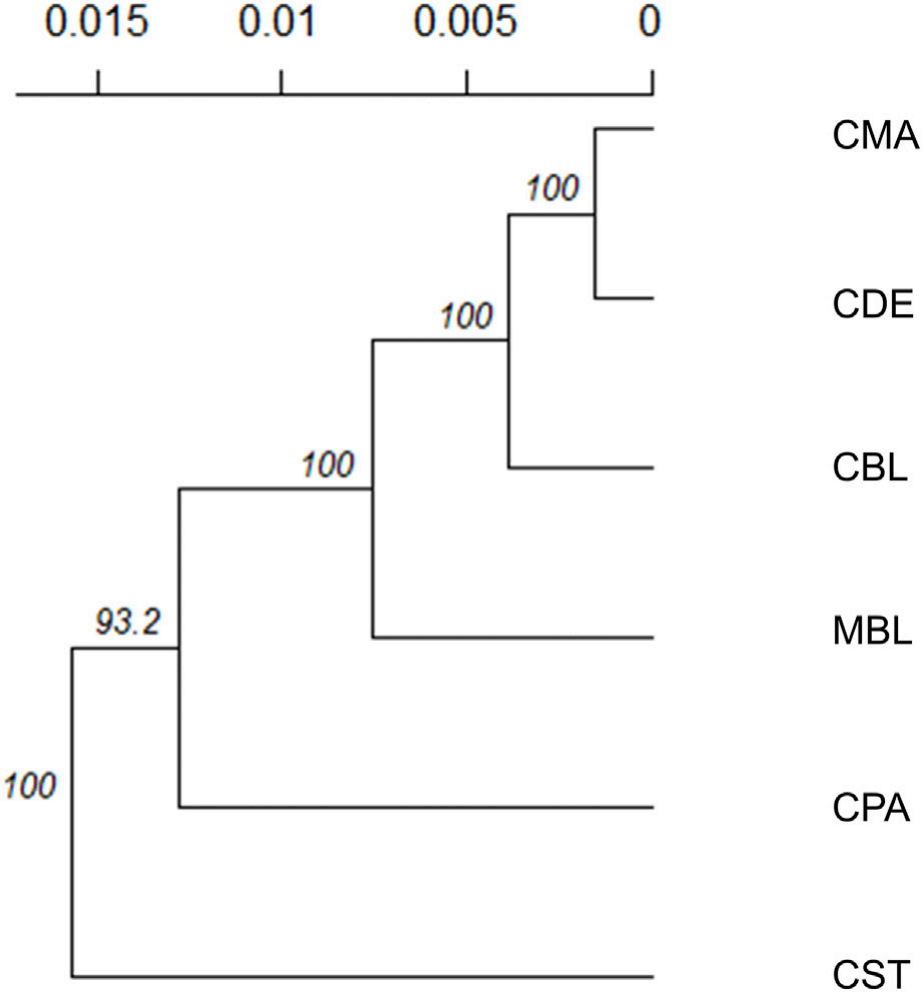
Unrooted consensus tree showing the genetic relationships among the six color types using the unweighted pair group method and the unbiased Nei’s genetic distance. The values at the nodes are the percentages of bootstrap values from 1,000 replications of resampling. The *x*-axis represents the genetic distances between color types. MBL = black color type mink in Millbank Fur Farm; CBL, CDE, CMA, CPA, and CST are black, demi, mahogany, pastel, and stardust color type mink in the Canadian Center for Fur Animal Research, respectively.

Weir and Cockerham’s Fst and Nei’s genetic distances among six color types are shown in Table 3. None of the Fst values between the two color types were larger than 0.1, and none of the Nei’s genetic distances between the two color types were larger than 0.06. The lowest Fst (0.006) and Nei’s genetic distance (0.003) were found between CMA and CDE. The CPA and CST showed the highest Fst (0.096) and Nei’s genetic distance (0.053). To examine the genetic relationship among various color types, a phylogenetic tree was also constructed using the unweighted pair group method and Nei’s genetic distance (Figure 3). The phylogenetic tree revealed two main clusters, with CST in one cluster and CDE, CPA, CMA, CBL, and MBL in the second cluster. Meanwhile, CDE and CMA were assigned to the subgroup with the least genetic distance.

**TABLE 3:** Estimation of Nei’s genetic distance (upper diagonal) and Weir and Cockerham’s Fst (lower diagonal) between various color types of American mink.

	CDE^a^	CPA^b^	CMA^c^	CST^d^	CBL^e^	MBL^f^
CDE	0	0.012	0.003	0.027	0.010	0.018
CPA	0.024	0	0.021	0.053	0.033	0.037
CMA	0.006	0.044	0	0.023	0.005	0.012
CST	0.042	0.096	0.033	0	0.018	0.035
CBL	0.021	0.068	0.010	0.024	0	0.015
MBL	0.040	0.081	0.028	0.063	0.035	0

^a^ Demi color type mink in the canadian center for fur animal research (CCFAR).

^b^ Pastel color type mink in CCFAR.

^c^ Mahogany color type mink in CCFAR.

^d^ Stardust color type mink in CCFAR.

^e^ Black color type mink in CCFAR.

^f^ Black color type mink in Millbank Fur Farm.

The results from AMOVA for various color types are shown in Table 4. The differentiation within color types represented the highest proportion of total molecular variation in the populations (91.6%). The variation among color types was significant (*p* < 0.05) but only represented 4.1% of the total molecular variation in the populations. The variation among farms was estimated to represent 4.3% of the total molecular variation in the populations but was not significant (*p* > 0.05).

**TABLE 4:** Analysis of molecular variance (AMOVA) in various color types of American mink.

Source of variation	Degrees of freedom	Sum of squares	Mean squared deviations	Variance components	Percentage of variation
Among farms	1	10.720	10.720	0.004	4.291
Among color types	4	4.425	1.106	0.004^*^	4.067
Within color types	2,967	279.795	0.094	0.094^*^	91.642
Total	2,972	294.940	11.920	0.102	100

^*^ *p* < 0.05.

### 3.3 Genetic structure and admixture patterns

**FIGURE 4:**
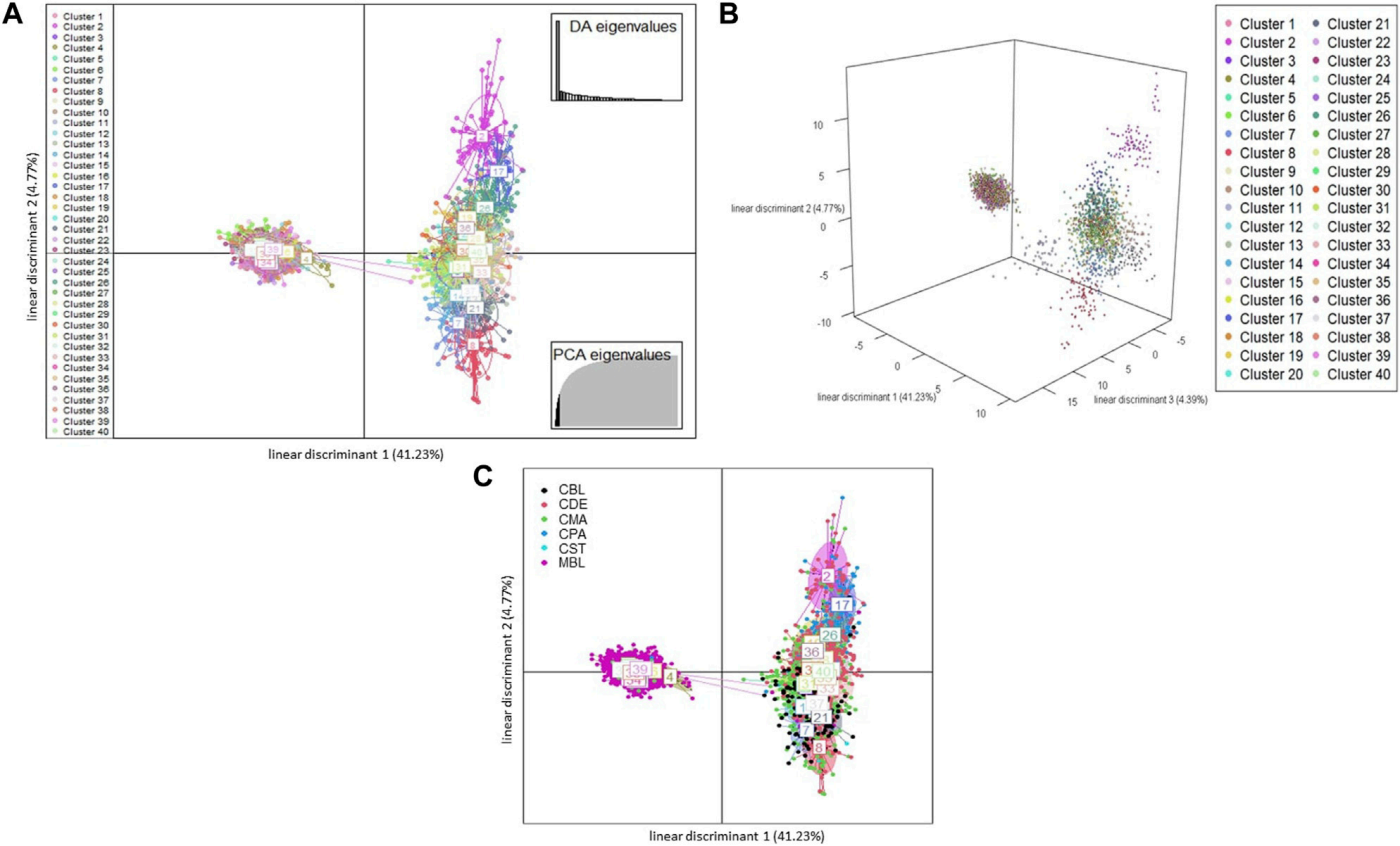
The scatterplots of discriminant analysis of principal components Each ellipse represents a cluster, and each dot represents an individual. Different clusters are separated by colors. MBL = black color type mink in Millbank Fur Farm; CBL, CDE, CMA, CPA, and CST are black, demi, mahogany, pastel, and stardust color type mink in CCFAR, respectively. **(A)** The scatterplot of the first two linear discriminants (*x* and *y*-axis, respectively), which explained 41.23% and 4.77% of the variation, respectively. Individual dot is a given color based on which cluster the individual is grouped to; **(B)** The 3D scatterplot of the first three linear discriminants (x, y, and *z*-axis, respectively), which explained 41.23, 4.77, and 4.39% of the variation, respectively. Individual dot is a given color based on which cluster the individual is grouped to; and **(C)** The scatterplot of the first two linear discriminants (*x* and *y*-axis, respectively). Different clusters are separated by colors and inertia ellipses labeled with a number. Individual dot is a given a color based on the individual color type.

**FIGURE 5:**
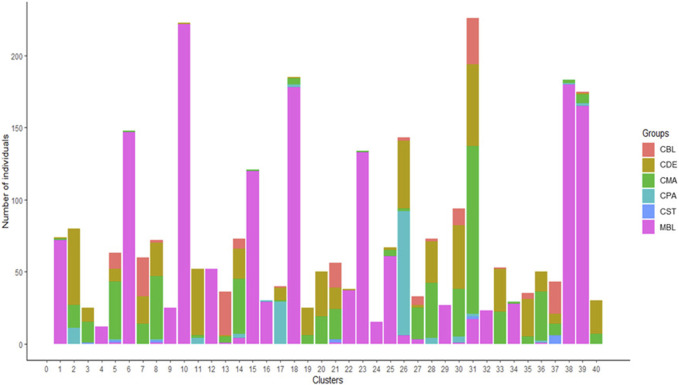
The number of individuals from various color types in 40 assigned clusters inferred by discriminant analysis of principal components. MBL = black color type mink in Millbank Fur Farm; CBL, CDE, CMA, CPA, and CST are black, demi, mahogany, pastel, and stardust color type mink in the Canadian Center for Fur Animal Research, respectively.

**FIGURE 6:**
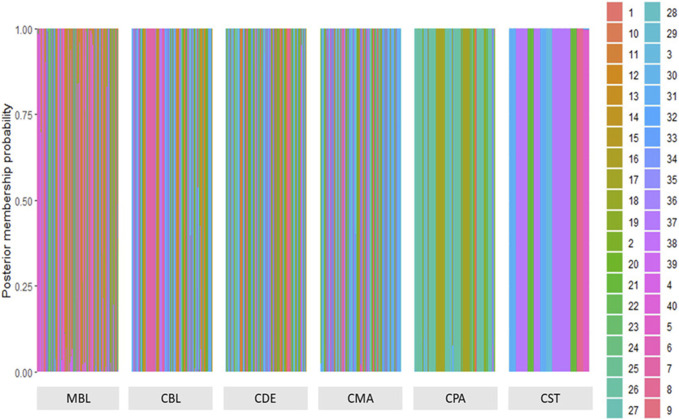
The probability of membership of each sample in the 40 assigned clusters inferred by discriminant analysis of principal components. MBL = black color type mink in Millbank Fur Farm; CBL, CDE, CMA, CPA, and CST are black, demi, mahogany, pastel, and stardust color type mink in the Canadian Center for Fur Animal Research, respectively.

A total of 123 principal components were retained for DAPC analysis based on the result of the α-score optimization analysis (Supplementary Figure S1). Sequential K-means clustering and the BIC indicated an optimum of 40 clusters in the studied populations (Supplementary Figure S2), and the DAPC showed 40 clusters in Figure 4; Figures 4A, B present the scatterplots of the first two linear discriminants and the first three linear discriminants for all samples, respectively; Figure 4C shows the distribution of various color types of mink in these 40 clusters in the scatterplot of the first two linear discriminants. The number of individuals from different color types in each cluster is shown in Figure 5. Eighteen clusters concentrated on the left side of the *y*-axis, and most MBL individuals were grouped into those clusters (Figures 4A, C). Twenty-two clusters spread on the right side of the *y*-axis, and most of the CCFAR individuals were grouped into these clusters (Figures 4A, C). Compared with the clusters on the right side of the *y*-axis, where most of the CCFAR individuals were located, the clusters on the left side of the *y*-axis, where most of the MFF individuals were located, were more concentrated in a smaller area and more overlapped with each other. The three-dimensional scatterplot separated the clusters located on the right side of the *y*-axis of the DAPC scatterplot of the first two linear discriminants more widely but not the clusters located on the left side of the *y*-axis (Figure 4B). MBL individuals were classified into 28 clusters and were the dominant color type in 18 of those clusters. Individuals in CDE, CPA, CMA, CST, and CBL color types were classified into 28, 15, 31, 6, and 16 clusters, respectively, and were not the dominant color type in those clusters (Figure 5). Additionally, the individual posterior membership probabilities to different clusters are presented in Figure 6. All the color types were largely admixed with several different clusters.

**FIGURE 7:**
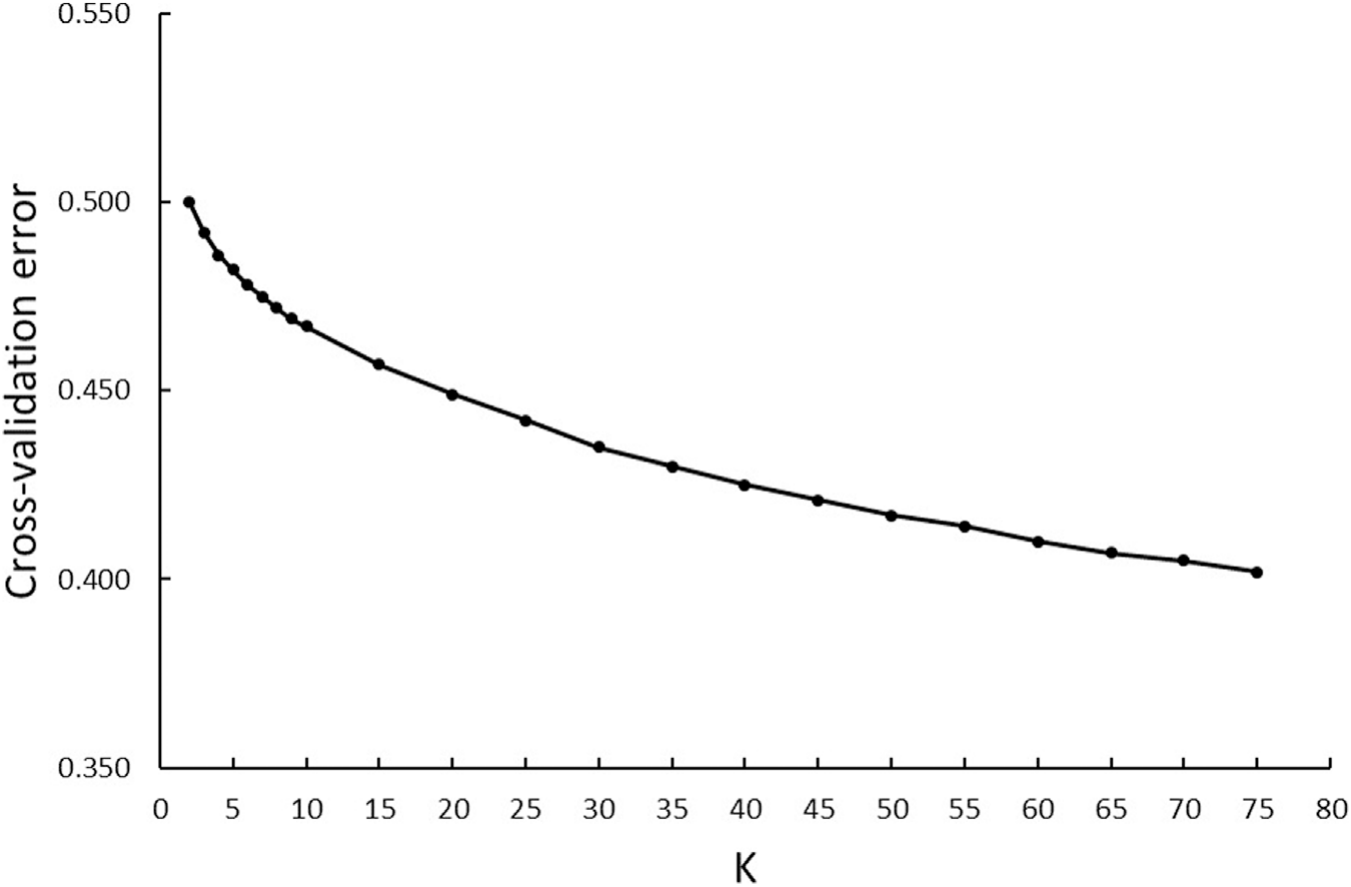
ADMIXTURE analyses of six color types American mink with cross-validation error plot for K-values from 2 to 75.

**FIGURE 8:**
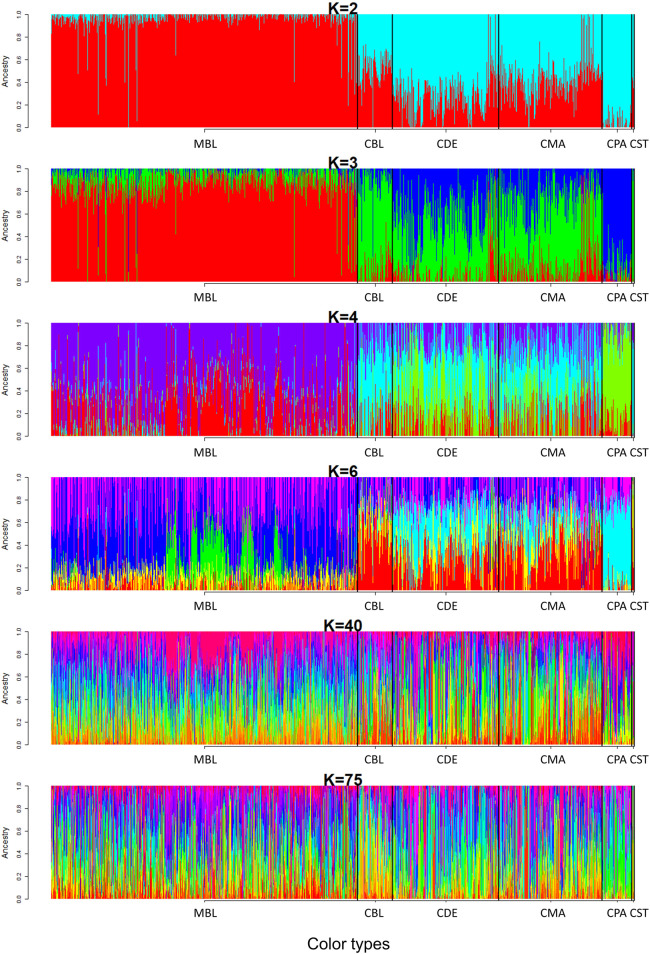
Admixture pattern of six color types American mink at K = 2, 3, 4, 6, 40, and 75. MBL = black color type mink at Millbank Fur Farm; CBL, CDE, CMA, CPA, and CST are black, demi, mahogany, pastel, and stardust color type mink in the Canadian Center for Fur Animal Research, respectively.

A model-based maximum likelihood approach was used to infer population structure at different K levels (Figures 7, 8). The CV error was markedly reduced with each increase in K until *K* = 4. Hereafter, the CV error gradually decreased with an increasing K, but the differences in CV error between adjacent Ks were less and less. The lowest CV was found when *K* = 75 within the range of K values that we tested (Figure 7). The ADMIXTURE runs for *K* = 2, 3, 4, 6, 40, and 75 are shown in Figure 8. The results indicated that the most likely partition was for *K* = 3, based on visual inspection of the admixture plots. The ideal method to define the number of K should be based on the CV error, but the CV error in this study kept decreasing with increased K (Figure 7). Visual inspection of admixture plots was used to define the best K according to the other studies (Mujibi et al., 2018; Lal et al., 2021). At *K* = 2, a clear distinctness between MBL and CPA was found. The rest of the color types were admixed with two clusters. At *K* = 3, the study populations showed a relatively distinguishable distinctness between CCFAR and MFF populations. Most MFF individuals were assigned to one cluster (average 85.26% on ancestry fractions), and the other two clusters were dominant in the CCFAR population. The CBL (average of 77.51% on ancestry fractions) and CPA (average of 79.13% on ancestry fractions) became distinct clusters within the CCFAR population and the two main genetic compositions in the CCFAR population. In the meantime, CDE and CMA were admixed with the three clusters and seemed to share a similar admixture pattern. When *K* = 4 and 6, except for the CPA color type, where one cluster seemed like the dominant cluster in this color type, all other color types were admixed with at least two clusters. In the meantime, the CCFAR population showed a higher level of admixture than the MFF population. When *K* = 40 and 75, no obvious distinction in ADMIXTURE among various color types was found, and all color types were admixed with several clusters (Figure 8).

## 4 Discussion

The average *r* ^2^ between adjacent SNPs on all chromosomes for various color types were in the range of 0.373–0.406. The estimated average *r* ^2^ between adjacent SNPs was higher than the estimates from previous studies (Karimi et al., 2020; Karimi et al., 2021). The average *r* ^2^ was estimated to be 0.30 using 13,321 SNPs extracted from 99 scaffolds with GBS data (Karimi et al., 2020). The average *r* ^2^ was estimated to range from 0.280 to 0.366 for various color types using 100,000 SNPs extracted from 51 scaffolds with the WGS data (Karimi et al., 2021). The different SNP marker densities, sample sizes, data resources, using incomplete scaffold-based vs complete chromosome-based reference genomes, and population structures among the studies are the potential causes leading to these discrepancies. The *r* ^2^ > 0.2 is considered the minimum threshold value for genomic selection to achieve an accuracy of >0.85 (Meuwissen et al., 2001; Hayes et al., 2009; Samorè and Fontanesi, 2016). In the current study, the average *r* ^2^ of CCFAR and MFF populations decreased to <0.2 when the inter-marker interval reached larger than 350 kb and 650 kb, respectively. These estimates indicated that the minimum marker density for conducting genomic selection at acceptable accuracy for the CCFAR population is about 7,700 SNPs (2.68 Gb/350 kb, where 2.68 Gb (Karimi et al., 2022) is the size of American mink genome assembly) and about 4,200 SNPs (2.68 Gb/650 kb) for the MFF population. For GWAS, *r* ^2^ > 0.3 is commonly used as the ideal threshold LD to obtain sufficient power and accuracy (Ardlie et al., 2002; Wang et al., 2018; Wu et al., 2019; Zhang Y. et al., 2021). The *r* ^2^ was estimated to be more than 0.3 when the marker distances were less than 125 kb and 225 kb for CCFAR and MFF populations, respectively, which indicated approximate 22,000 (2.68 Gb/125 kb) and 12,000 (2.68 Gb/225 kb) SNPs are necessary to conduct GWAS in CCFAR and MFF populations, respectively. It has been noted that more markers are needed to perform adequate genomic studies in admixed populations (Toosi et al., 2010; Thomasen et al., 2013; Brito et al., 2015; Karimi et al., 2021). Thus, the higher level of admixture in the CCFAR population may be the reason for the required higher marker density for conducting genomic selection in this population compared to the MFF population. Using GBS data, Karimi et al. (2020) suggested the density of 60,000 SNPs and 120,000 SNPs, which were all higher than the estimates in the current study, are required for conducting genomic selection and GWAS in black American mink, respectively. Karimi et al. (2021) suggested a larger number of SNPs to conduct genomic selection (120,000 for CCFAR and 24,000 for MFF) and GWAS (240,000 for both farms) by using WGS data of 100 American mink from the same population. The different estimates of *r* ^2^ between the current study and previous studies (Karimi et al., 2020; Karimi et al., 2021) are the causes for the different suggested marker densities of conducting genomic selection and GWAS in American mink.

In this study, the *Ne* at five generations ago was estimated to be 76 and 91 for CCFAR and MFF populations, respectively. In Spain, the *Ne* of American mink in six locations ranged from 7.2 to 34.8 using information from ten polymorphic microsatellite loci (Lecis et al., 2008). On Swedish coasts, depending on the geographical location of the sampling, the *Ne* of American mink was estimated to be from 17.5 to 70.8 using genotypes from 21 microsatellite markers (Zalewski et al., 2016). The *Ne* at five generations ago was estimated to be 116 for black American mink, which was also higher than the estimates in this study, using SNP data obtained from the GBS data (Karimi et al., 2020). Compared with this study, Karimi et al. (2021) estimated a higher *Ne* at five generations ago (99) for CCFAR and a lower *Ne* at five generations ago (50) for MFF using SNP information extracted from the WGS data of 100 mink. The estimates of the current study indicated that the *Ne* declined more rapidly from 5 to 50 generations ago for the CCFAR and from 5 to 100 generations ago for the MFF population, which coincided with the time when the farms were established. The CCFAR was established about 40 years ago (1984), and the MFF was founded over 90 years ago (1930). These trends were different from those estimated by using WGS data (Karimi et al., 2021) and GBS data (Karimi et al., 2020), where the decline of *Ne* was more rapid between 150 and 200 generations ago. The different estimations of LD patterns among studies are the cause leading to the different *Ne* estimates. The *Ne* of the CCFAR population decreased more rapidly than the MFF population in more recent generations (5–50 generations ago). This may be related to different breeding managements and strategies, population genetic backgrounds, and populations sizes (MFF has larger population size than CCFAR) in these two farms.

Both Fst (less than 0.1) and Nei’s genetic distances (less than 0.06) among various color types were low, which indicated the low genetic differentiation among various color types. This was in agreement with the AMOVA results, where the variation within color types explained 91.6% of total molecular variation, while the variation among color types only explained 4.1% of the total molecular variation in the populations. Compared with other color types, CST had the farthest genetic distances (Nei’s genetic distance values) with CDE, CPA, CMA, and MBL color types. This was in agreement with the result from the phylogenetic tree, which separated CST into a separate cluster from other color types. The Fst and Nei’s genetic distances among various color types were estimated in the range of 0.015–0.124 and 0.013 to 0.065, respectively, using WGS data (Karimi et al., 2021). They were all slightly higher than the estimates from this study (Fst ranged from 0.006 to 0.096, and Nei’s genetic distance ranged from 0.003 to 0.053). The overall Fst among seven color types of American mink from 14 different geographical locations in Poland and Denmark was estimated to be 0.08 by Thirstrup et al. (2015) using information from 194 SNPs generated from the restriction-site associated DNA sequencing data, which was also higher than the overall Fst (0.041) among six color types in the study. In southern Chile, the Fst among 153 mink obtained from 12 locations were estimated to be in the range from 0.017 to 0.364, which was also higher than the estimates in this study (0.006–0.096), using genotypic data from 11 polymorphic microsatellite loci (Mora et al., 2018).

The mink of the six color types were differentiated into 40 clusters using multivariate DAPC analysis in this study. Individuals within the same color type were divided into clusters ranging from six to 31 (Figures 5, 6), which indicated the existence of genetic differentiation among mink within the same color. These results were in agreement with the result from AMOVA (Table 4), where the variation within color types represented the majority of the total molecular variation in the populations, and the high level of nucleotide diversity within each color type (Table 1). In the meantime, 97% (1,526 individuals) of MBL individuals and 97% (1,378 individuals) of CCFAR individuals were grouped into the clusters located on the left and right side of the *y*-axis of the DAPC scatterplot of the first two linear discriminants (Figure 4C), respectively. These results indicated that the DAPC analysis was able to separate CCFAR and MFF populations by the first linear discriminant (explained 41.23% of the variance). However, DAPC analysis was not able to further differentiate the clusters within a farm as the clusters on the same side of the *y*-axis of the DAPC scatterplots were overlapped, and the distances between clusters were minimal (Figure 4). This may be caused by high gene flow and admixture events in recent generations of the studied populations because there was no regular mating system on both farms, and animals were mostly selected based on their phenotypic performances without considering their color types. Additionally, the clusters, where most of the CCFAR individuals were located, were more dispersed in a wider area and less overlapped with each other on the DAPC scatterplots compared with the clusters, where most of the MFF individuals were located (Figure 4), indicating CCFAR population may have a higher level of admixture than MFF population. These results were in agreement with the admixture patterns we observed when *K* = 2, 3, 4, and 6, where the CCFAR population showed a higher level of admixture than the MFF population (Figure 8). This may be related to the introduction of breeders from different farms within after the Aleutian disease outbreak in CCFAR in 2013 (the generation interval is 1 year in CCFAR), as multiple breeder sources may result in higher levels of admixture (Verhoeven et al., 2011; Parker et al., 2017; Karaman et al., 2021). Most of the mink in the barn were dead or culled at that time when Aleutian disease occurred. Thus, within 3 years of the disease outbreak, about 150 mink (120 dam and 30 sires) from six farms were introduced and used as breeders in the breeding season at CCFAR, which might lead to a higher admixture level in the population compared with the MFF population. The populations (CCFAR and MFF) were clustered into only three groups in DAPC analysis using WGS data from 100 individuals (Karimi et al., 2021). Compared to the DAPC results from this study, the MFF population was not clearly differentiated from the CCFAR population, and individuals within the same color tended to be grouped in the same cluster instead of several different clusters. The marker densities (100 k vs 24 k) and sample sizes (100 vs. 2,973) are the possible reasons leading to the differences. The DAPC analysis differentiated 205 American mink in three different areas of Sweden into five clusters using the genotypic data from 21 microsatellites (Zalewski et al., 2016). The five clusters clearly differentiated the individuals from different study areas, indicating that geographical distribution was one of the critical factors in differentiating American mink. In our study, the geographical distribution might also play an important role in differentiating mink from two populations because the CCFAR and MFF populations were clearly separated by the first linear discriminant in the DAPC scatterplot (Figure 4).

In this study, three ancestral genetic groups were considered to delineate the studied populations’ genetic structure based on the change of CV error against K and visual inspection of the admixture plots. The change in CV error against K (Figure 7) indicated that the improvement in model fitness started to decrease between *K* = 3 and *K* = 5, suggesting that *K* = 3 may be the best cluster number that describes the studied populations. Compared to the admixture plots when *K* = 2, 4, 6, 40, and 75, the admixture plot when *K* = 3 seemed to describe the genetic structure of studied populations better. Similar to the DAPC results, when *K* = 3, a distinguishable distinctness between CCFAR and MFF populations was observed in the admixture plot, which further illustrated geographical distribution as a critical factor in differentiating American mink. The CCFAR population showed a higher level of admixture than the MFF population, which might be caused by the use of breeders from multiple sources in CCFAR (introduced breeders from six different farms after the Aleutian disease outbreak) and a relatively single breeder source in MFF. Meanwhile, MBL and CBL were clearly identified with a distinct ancestral population suggesting that these two black color types derive from different ancestral populations and color type might not be a reliable indicator to differentiate American mink. Within the CCFAR population, CBL and CPA had their own clusters, and CDE, CMA, and CST showed noticeable admixtures of these two clusters. Many color types in American mink are exclusively line-bred because many color types are recessive to the standard brown color type, and the rest are blended (Shackelford, 1948; Joergensen, 1985; Nes et al., 1988). For example, mahogany is achieved by breeding the black and standard brown color types (Joergensen, 1985). This could explain the admixture patterns of CMA and CDE in this study since these are visually very close color types, and CPA is one of the dominant brown color types. In the meantime, CDE and CMA seemed to share a similar admixture pattern, which indicated these two color types might share a similar genetic structure. Several results from this study also supported the point that CDE and CMA had a similar genetic structure: 1) CDE and CMA showed the closest genetic distance (lowest Nei’s and Fst values) among all color types (Table 3); 2) the phylogenetic tree grouped CDE and CMA into the same subgroup (Figure 3); 3) CDE and CMA showed a similar trend in LD and *Ne* decay (Figures 1, 2); and 4) most of CDE and CMA individuals appeared in the same clusters generated from DAPC analysis (Figure 5). These results further illustrated the color type may not be a reliable indicator to differentiate American mink populations. The admixture patterns from this study are similar to the estimated admixture patterns of American mink in Canada in previous studies. The admixture patterns of farmed and wild American mink in Ontario, Canada, were investigated by Kidd et al. (2009) using the data from 10 microsatellite loci. In their studied farm populations, mink in black and pastel color types had their own groups, and mink in mahogany color type were mixed with several groups (Kidd et al., 2009), which were in the same patterns as this study. The admixture patterns of the current studied populations were also investigated in the small sample size using WGS data (Karimi et al., 2021). Similar to the results from this study, when *K* = 3, individuals in black and pastel color types had their own groups, and individuals in demi and mahogany color types were mixed with those three groups (Karimi et al., 2021).

Genetic structure and admixture pattern analyses conducted in this study did not detect clear genetic distinctions among the mink of various color types. Two potential reasons could lead to these results. The studied individuals were sampled from only two farms, which might make the samples not the ideal sample structures to reveal the population structures. Thus, future studies should consider including animals of various color types from wild and more geographically distributed farms. In the meantime, it has been noted that a larger number of markers may be needed to resolve population genomics studies when the genetic distance (Fst) between the populations is low (Patterson et al., 2006). Thus, future studies could impute the SNP genotypes to WGS to obtain larger marker density to further investigate the genetic structure and admixture pattern of the studied populations.

## 5 Conclusion

In this study, 2,973 animals from two farms and their genotypes obtained from the first developed medium-density SNP panel for American mink were used to investigate their LD patterns and genetic structure in various color types. The estimated LD patterns suggested that 7,700 and 4,200 SNPs are the minimum marker densities to conduct genomic selection programs in CCFAR and MFF populations, respectively. The results from genetic distances and diversity analyses indicated genetic differentiation among various color types was low, and most of the genetic variation occurred within color types rather than between color types. Three ancestral genetic compositions were considered the most appropriate number of ancestral genetic compositions to delineate the populations’ genetic structure. The black (both CCFAR and MFF) and pastel color types seemed to have their own ancestral clusters, and demi, mahogany, and stardust were admixed with the three ancestral genetic compositions. Additionally, mink in demi and mahogany color types seemed to have a similar admixture pattern, but further study is needed. The genetic structure and admixture pattern of mink of various color types and within the same color type were not clearly identified in this study. Thus, future studies with samples from wider geographically distributed locations and higher marker density are needed to differentiate the mink within the same color type.

This study provided useful information for conducting genomic evaluations in the mink industry using genotypes from the medium-density SNP panel. The mink industry faces several challenges caused by the COVID-19 pandemic, industry downturn, and decreasing market demand. Improving production efficiency through advanced genomic approaches could help the mink industry meet these challenges. The LD patterns and genetic structures obtained from the first SNP panel for American mink would provide the essential information to implement the SNP panel in genomic studies of American mink.

## Data Availability

The datasets presented in this article are not suitable for public deposition due to legal restrictions and are therefore available upon request for verification of our results. Requests to access the datasets should be directed to YM.
