# Corrigendum: Population genomics of American mink using genotype data

**DOI:** 10.3389/fgene.2023.1221683

**Published:** 2023-05-19

**Authors:** Guoyu Hu, Duy Ngoc Do, Ghader Manafiazar, Alyson A. Kelvin, Mehdi Sargolzaei, Graham Plastow, Zhiquan Wang, Younes Miar

**Affiliations:** ^1^ Department of Animal Science and Aquaculture, Dalhousie University, Truro, NS, Canada; ^2^ Vaccine and Infectious Disease Organization (VIDO), University of Saskatchewan, Saskatoon, SK, Canada; ^3^ Department of Pathobiology, University of Guelph, Guelph, ON, Canada; ^4^ Select Sires Inc., Plain City, OH, United States; ^5^ Livestock Gentec, Department of Agricultural, Food and Nutritional Science, University of Alberta, Edmonton, AB, Canada

**Keywords:** American mink, effective population size, linkage disequiblibrium, population genomics, genotypes

A correction has been made to **Acknowledgments**. **Paragraph 1**. This sentence previously stated:

“We also thank the Canadian Center for Fur Animal Research and Millbank Fur Farm staff for collecting and providing the data.”

The corrected sentence appears below:

“We also thank the Canadian Center for Fur Animal Research and Millbank Fur Farm staff for collecting and providing the data and the members of Miar Lab (https://miarlab.ca/personnel) who helped in the molecular lab and farm.”

The authors apologize for this error and state that this does not change the scientific conclusions of the article in any way. The original article has been updated.

