# Supplementary material for: Population genomics of American mink using genotype data
Source: Front Genet. 2023 May 9;14:1175408. doi: 10.3389/fgene.2023.1175408 (PMC10234291; doi:10.3389/fgene.2023.1175408)
Supplement: Supplementary file 1 [file Image2.pdf]

Supplementary Material

Population Genomics of American Mink Using Genotypes Data

Guoyu Hu, Duy Ngoc Do, Ghader Manafiazar, Alyson A. Kelvin, Mehdi Sargolzaei, Graham Plastow, Zhiquan Wang, and Younes Miar*

*** Correspondence:** Younes Miar, miar@dal.ca

1 Supplementary Figures

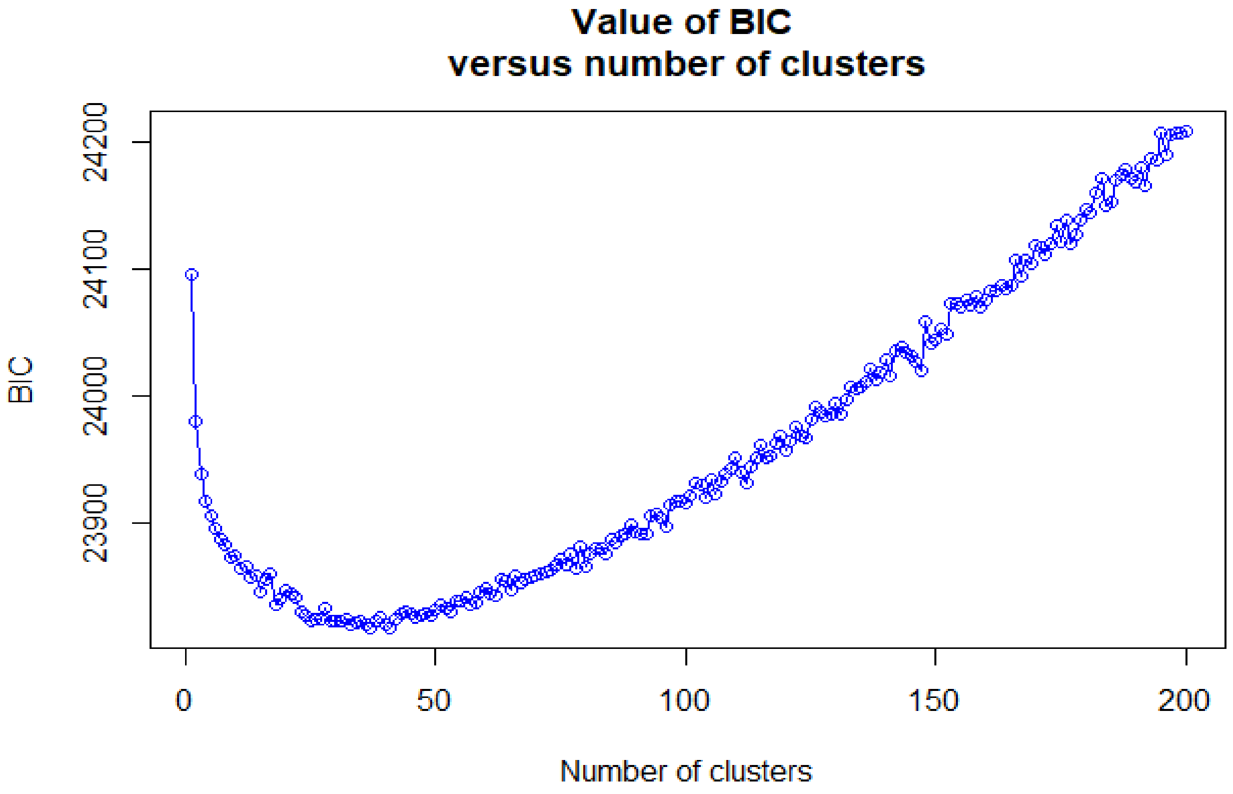

Supplementary Figure 2. Population genetic clustering results based on the Bayesian Information Criterion (BIC) in relation to the number of clusters identified by the find.cluster function in DAPC analysis.
