# Supplementary material for: Population genomics of American mink using genotype data
Source: Front Genet. 2023 May 9;14:1175408. doi: 10.3389/fgene.2023.1175408 (PMC10234291; doi:10.3389/fgene.2023.1175408)
Supplement: Supplementary file 2 [file Image1.pdf]

*Supplementary Material*

# Population Genomics of American Mink Using Genotypes Data

**Guoyu Hu, Duy Ngoc Do, Ghader Manafiazar, Alyson A. Kelvin, Mehdi Sargolzaei, Graham Plastow, Zhiquan Wang, and Younes Miar***

*** Correspondence:** Younes Miar, miar@dal.ca

## 1 Supplementary Figures

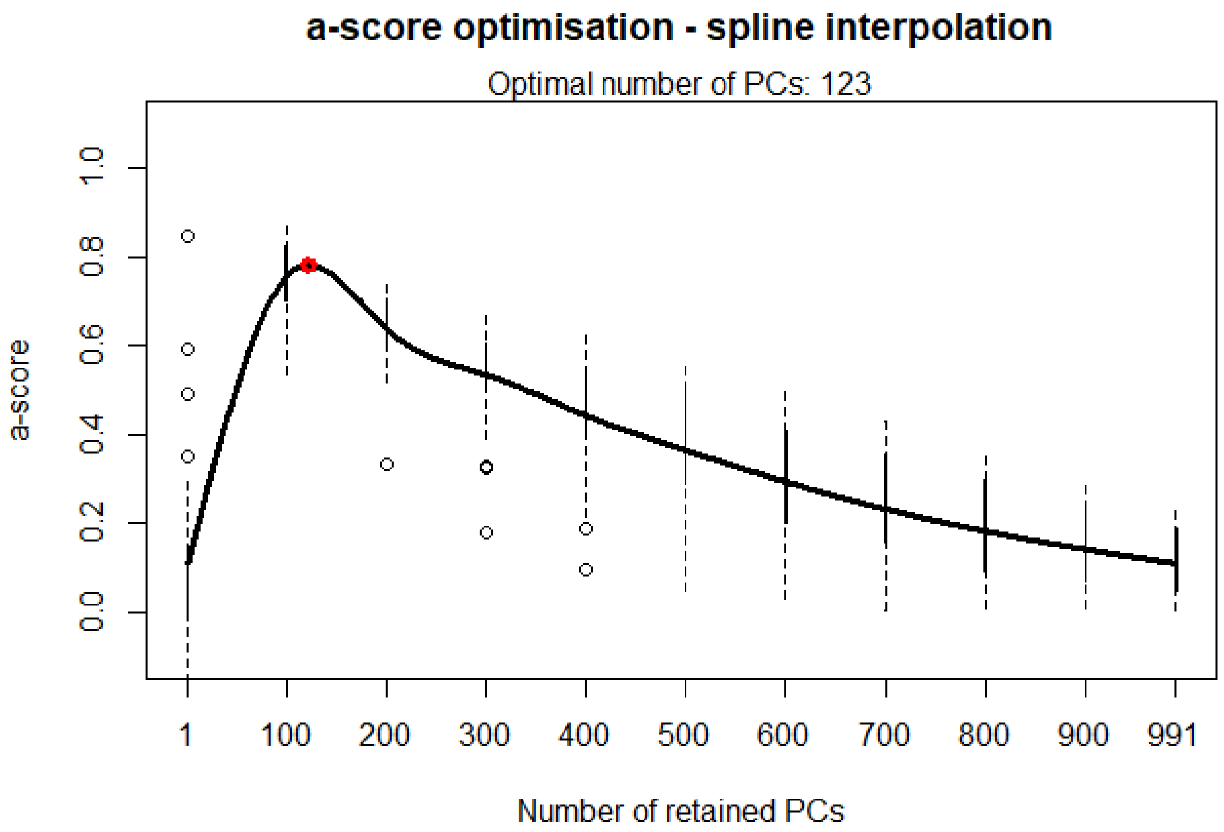


**Supplementary Figure 1.** Optimization α-score graph.
